# Etiological spectrum of persistent fever in the tropics and predictors of ubiquitous infections: a prospective four-country study with pooled analysis

**DOI:** 10.1186/s12916-022-02347-8

**Published:** 2022-05-02

**Authors:** Emmanuel Bottieau, Lukas Van Duffel, Sayda El Safi, Kanika Deshpande Koirala, Basudha Khanal, Suman Rijal, Narayan Raj Bhattarai, Thong Phe, Kruy Lim, Deby Mukendi, Jean-Roger Lilo Kalo, Pascal Lutumba, Barbara Barbé, Jan Jacobs, Marjan Van Esbroeck, Nikki Foqué, Achilleas Tsoumanis, Philippe Parola, Cedric P. Yansouni, Marleen Boelaert, Kristien Verdonck, François Chappuis

**Affiliations:** 1 Department of Clinical Sciences, Institute of Tropical Medicine, Antwerp, Belgium; 2 Infectious Diseases Operative Unit, Santa Maria delle Croci Hospital, AUSL Romagna, Ravenna, Italy; 3 Faculty of Medicine, University of Khartoum, Khartoum, Sudan; 4 B. P. Koirala Institute of Health Sciences, Dharan, Nepal; 5 Sihanouk Hospital Center of HOPE, Phnom Penh, Cambodia; 6 Institut National de Recherche Biomédicale, Kinshasa, Democratic Republic of Congo; 7 Service de neurologie, Université de Kinshasa, Kinshasa, Democratic Republic of Congo; 8 Department of Microbiology, Immunology and Transplantation, KU Leuven, Leuven, Belgium; 9 IHU-Méditerranée Infection & Aix-Marseille University, Marseille, France; 10 JD MacLean Centre for Tropical Diseases, McGill University Health Centre, Montreal, Canada; 11 Department of Public Health, Institute of Tropical Medicine, Antwerp, Belgium; 12 Division of Tropical and Humanitarian Medicine, Geneva University Hospitals and University of Geneva, Geneva, Switzerland

**Keywords:** Persistent fever, Tropics, Leptospirosis, Enteric fever, Rickettsiosis, Malaria, Tuberculosis, HIV infection, Rapid diagnostic tests

## Abstract

**Background:**

Persistent fever, defined as fever lasting for 7 days or more at first medical evaluation, has been hardly investigated as a separate clinical entity in the tropics. This study aimed at exploring the frequencies and diagnostic predictors of the ubiquitous priority (i.e., severe and treatable) infections causing persistent fever in the tropics.

**Methods:**

In six different health settings across four countries in Africa and Asia (Sudan, Democratic Republic of Congo [DRC], Nepal, and Cambodia), consecutive patients aged 5 years or older with persistent fever were prospectively recruited from January 2013 to October 2014. Participants underwent a reference diagnostic workup targeting a pre-established list of 12 epidemiologically relevant priority infections (i.e., malaria, tuberculosis, HIV, enteric fever, leptospirosis, rickettsiosis, brucellosis, melioidosis, relapsing fever, visceral leishmaniasis, human African trypanosomiasis, amebic liver abscess). The likelihood ratios (LRs) of clinical and basic laboratory features were determined by pooling all cases of each identified ubiquitous infection (i.e., found in all countries). In addition, we assessed the diagnostic accuracy of five antibody-based rapid diagnostic tests (RDTs): Typhidot Rapid IgM, Test-it^TM^ Typhoid IgM Lateral Flow Assay, and SD Bioline Salmonella typhi IgG/IgM for *Salmonella* Typhi infection, and Test-it^TM^ Leptospira IgM Lateral Flow Assay and SD Bioline Leptospira IgG/IgM for leptospirosis.

**Results:**

A total of 1922 patients (median age: 35 years; female: 51%) were enrolled (Sudan, *n* = 667; DRC, *n* = 300; Nepal, *n* = 577; Cambodia, *n* = 378). Ubiquitous priority infections were diagnosed in 452 (23.5%) participants and included malaria 8.0% (*n* = 154), tuberculosis 6.7% (*n* = 129), leptospirosis 4.0% (*n* = 77), rickettsiosis 2.3% (*n* = 44), enteric fever 1.8% (*n* = 34), and new HIV diagnosis 0.7% (*n* = 14). The other priority infections were limited to one or two countries. The only features with a positive LR ≥ 3 were diarrhea for enteric fever and elevated alanine aminotransferase level for enteric fever and rickettsiosis. Sensitivities ranged from 29 to 67% for the three RDTs targeting *S.* Typhi and were 9% and 16% for the two RDTs targeting leptospirosis. Specificities ranged from 86 to 99% for *S.* Typhi detecting RDTs and were 96% and 97% for leptospirosis RDTs.

**Conclusions:**

Leptospirosis, rickettsiosis, and enteric fever accounted each for a substantial proportion of the persistent fever caseload across all tropical areas, in addition to malaria, tuberculosis, and HIV. Very few discriminative features were however identified, and RDTs for leptospirosis and *Salmonella* Typhi infection performed poorly. Improved field diagnostics are urgently needed for these challenging infections.

**Trial registration:**

NCT01766830 at ClinicalTrials.gov.

**Supplementary Information:**

The online version contains supplementary material available at 10.1186/s12916-022-02347-8.

## Background

As the incidence of malaria is decreasing in the tropics, research has focused on the proportional increase of other etiologies of acute febrile illness (AFI), including the most challenging subgroup of acute undifferentiated febrile illness, i.e., with no focal symptoms [1]. Many single-center studies have investigated the etiologies of acute fever in various tropical areas in the past decade [2–7], and multi-country fever etiology studies are ongoing, such as FIEBRE (“Febrile Illness Evaluation in a Broad Range of Endemicities”) [8].

In contrast to AFI, there is almost no research on community-acquired persistent fever, which can be defined as a febrile illness lasting for 7 days or more at the first medical evaluation. Although this clinical entity is not commonly reported as such in the literature, it represents an important subgroup of febrile illness in the tropics, usually excluded from AFI studies. The etiological spectrum of persistent fever is poorly defined but likely differs from that of acute fever. It may indeed be assumed that most self-limiting viral or bacterial diseases would have resolved after 7 days, as well as bacterial infections that are susceptible to first-line empirical antibiotic treatment. It is therefore expected that parasitic and fungal etiologies, including some neglected infectious diseases such as visceral leishmaniasis (VL) or human African trypanosomiasis (HAT), non-responsive bacterial infections, and non-infectious conditions are proportionally more frequent in the persistent fever syndrome. Consequently, this clinical entity is important for both first- and second-line clinicians and appears particularly challenging when resources are limited [9].

To address knowledge gaps in the etiological spectrum and diagnostic approach of persistent fever in the tropics, we set up a clinical and diagnostic study within a larger international research project called NIDIAG (“Better DIAGnosis of Neglected Infectious Diseases”; www.NIDIAG.eu). For this study, a set of 12 epidemiologically relevant infections assumed to cause persistent fever were targeted in priority, because they were considered as “severe and treatable,” meaning that prompt diagnosis and locally available therapy could prevent adverse outcome even in low-resource settings. These 12 target “not-to-miss” conditions were enteric fever, leptospirosis, rickettsiosis, relapsing fever, brucellosis, melioidosis, VL, HAT, amebic liver abscess, malaria, tuberculosis, and HIV.

In addition to the three well-established ubiquitous conditions (i.e., malaria, tuberculosis and HIV), three other priority infections, i.e., enteric fever (due to *Salmonella enterica* Typhi or *S.* Paratyphi), leptospirosis, and rickettsiosis (spotted fever, typhus or *Orientia* groups), were expected to be found in most of the study sites, as AFI studies have shown all three to be endemic throughout the tropics [10]. While the field diagnosis of the former three infections has improved a lot in the past decade, the latter three bacterial infections remain notoriously difficult to diagnose in low-resource settings. The aims of this study were to determine the frequency of the conditions causing persistent fever in the tropics, with a focus on the priority infections, and to identify by pooled analysis the clinical and laboratory predictors of the subset of ubiquitous infections. A secondary objective was to report on the diagnostic accuracy of five rapid diagnostic tests (RDTs) evaluated for some of these infections (three targeting *S.* Typhi infection and two leptospirosis).

## Methods

### Study design and setting

This was a prospective multicentric clinical and diagnostic study embedded in the NIDIAG project, which was launched in 2010 by a consortium gathering three African, four Asian, and six European institutions. The overall aim of the NIDIAG project was the diagnosis of (neglected) infectious diseases in resource-poor settings by making the best possible use of existing assays. More specifically, the project focused on the timely detection of priority “severe and treatable” infectious diseases and on the development and evaluation of pathogen-specific RDTs. Three challenging clinical syndromes were investigated: persistent fever, neurological disorders, and persistent digestive syndrome (the latter two syndromes are reported elsewhere) [11, 12]. The persistent fever study took place in six study sites located in two African (Sudan and Democratic Republic of Congo, DRC) and two Asian (Nepal and Cambodia) countries: a rural hospital in Gedaref State, Sudan; a rural district hospital and an outpatient health center in Mosango, province of Kwilu, DRC; a rural district hospital in Dhankuta and a university hospital in Dharan, Nepal; and the referral-level Sihanouk hospital center of Hope in Phnom Penh, Cambodia: a rural hospital in Gedaref State, Sudan; a rural district hospital and an outpatient health center in Mosango, province of Kwilu, DRC; a rural district hospital in Dhankuta and a university hospital in Dharan, Nepal; and the referral-level Sihanouk hospital center of Hope in Phnom Penh, Cambodia. The NIDIAG sites were purposively chosen using the following criteria: presence of diverse etiologies of persistent fever (and in some cases also neurological and digestive disorders) including local as well as ubiquitous and neglected as well as common infectious diseases, a sufficiently large number of patients seeking care for the targeted syndromes, capacity to carry out a range of laboratory tests, experience with good clinical and good clinical laboratory practice (GCP and GCLP) and willingness to strengthen this further, and a history of successful collaboration among the investigators [13].

### Study participants

From January 2013 to October 2014, patients aged 5 years or older (in Cambodia 18 years or older, because the study site did not attend patients below 18 years) were enrolled if they presented with reported or documented fever of 7 days or more at one of the study sites. Although the intention was to enroll consecutive patients as much as possible, we limited the number of inclusions per day to four to five patients to avoid disrupting the local clinical and laboratory capacity. Patients were excluded if they already had a laboratory-confirmed diagnosis at the time of consultation, if they were already admitted since more than 48 h for another reason or if they presented with hemodynamic and/or respiratory instability and required immediate intensive care. After enrolment, participants were clinically evaluated and blood and urine samples were systematically collected for the diagnostic workup and evaluation of index RDTs. Treatment was provided after the initial diagnostic assessment according to local protocols. Follow-up took place within 1 month after inclusion at the study sites to allow paired serology, and after 3 months for final assessment of outcome, by phone call or site visit.

### Diagnostic procedures

First-line laboratory analyses consisted of basic hematology, biochemistry, and urinalysis at inclusion. Imaging (usually only ultrasound and X-ray) was performed when available and on clinical indication. For the 12 target priority infections, confirmatory diagnostic tests were performed either on site (blood culture, smear examination for parasites and mycobacteria, routine programmatic disease-specific RDTs,…) or later in reference laboratories (molecular and/or serological analysis of cryopreserved paired sera and urine) in the study countries or in Europe (Institute of Tropical Medicine, ITM, Antwerp, Belgium; Institut Hospitalier Universitaire, IHU, Méditerranée Infection, Marseille, France), as described in the Additional file 1, Table S1: Diagnostic ascertainment of NIDIAG target priority infections. The diagnosis of non-priority conditions such as focal infections (i.e., pneumonia, urinary tract infection,…) and “non-severe” (self-limiting) or “non-treatable” conditions (in a low-resource setting) was based on pre-established harmonized clinical case definitions or post-hoc diagnostic ascertainment by an expert panel, to ensure consistency across the sites (described in Additional file 1, Table S2: Case ascertainment of conditions not targeted by the NIDIAG workup).

Briefly, the reference diagnosis of the six ubiquitous priority infections was established as follows: positive results of the serial programmatic HIV-RDTs for HIV infection; microscopic examination or molecular assays for tuberculosis, or strict clinical case definitions; parasite-based diagnosis for malaria; isolation of *Salmonella enterica* Typhi (typhoid fever) or *S.* Paratyphi A or B (paratyphoid fever) in blood cultures for enteric fever; either a positive PCR assay in blood or urine or the appearance or four-fold rise of specific antibodies in acute/convalescent sera by the microagglutination test (MAT) for confirmation of leptospirosis (the diagnosis being considered as probable in case of a positive antibody test on single serum); and either a positive PCR on serum or seroconversion/antibody rise by indirect immunofluorescence assay for confirmation of rickettsiosis (probable diagnosis if positive IgM antibody on single acute serum).

Based on a previous literature review [14], three commercial antibody-based index RDTs for the diagnosis of *Salmonella* Typhi infection were evaluated in all the study sites: Typhidot Rapid IgM (Reszon Diagnostics International, Malaysia), Test-it^TM^ Typhoid IgM Lateral Flow Assay (Life Assay Diagnostics [Pty] Ltd, South Africa), and SD Bioline Salmonella typhi IgG/IgM (Standard Diagnostics, Republic of Korea). The latter assay, a three-band test, was considered positive if any line (IgM or IgG) was visible. Two commercial antibody-based index RDTs for the diagnosis for leptospirosis were also evaluated: Test-it^TM^ Leptospira IgM Lateral Flow Assay (Life Assay Diagnostics [Pty] Ltd, South Africa) and SD Bioline Leptospira IgG/IgM (Standard Diagnostics, Republic of Korea). The latter assay was also considered positive if any line (IgM or IgG) was positive. No index RDT was studied for rickettsiosis. The staff performing the reference testing was blinded to the clinical information and RDT results. The laboratory technicians performing the RDTs were not aware about the clinical suspicion or any reference test results.

### Endpoints and data analysis

There was no formal and homogenous sample size calculation per country since this study was exploratory, and we expected to find important variation across countries in disease profile and level of utilization of care facilities. The target size for the whole NIDIAG cohort of persistent fever was however set at 2000 participants, with at least 300 inclusions per country and a period of enrolment in each country of at least one year (to capture important seasonal variations).

We first described the frequency of the diagnoses established during the NIDIAG study, with a focus on the priority ubiquitous infections, observed in at least three countries. If a participant was diagnosed with more than one priority condition, only confirmed diagnoses were retained for the analysis. Next, using standard formulas, we determined the sensitivity, specificity, positive and negative likelihood ratios (LR+ and LR−), and predictive values of relevant clinical and laboratory features as well as index RDTs for the respective priority infections within the whole cohort. We excluded the cases with coinfections for the estimations of diagnostic accuracy because we considered that mixed etiologies could interfere with clinical presentation and RDT performance and therefore complicate the interpretation.

SAS 9.4 (SAS Institute, Cary NC) was used for statistical analyses, which were mainly descriptive. The results are reported in line with the Strengthening the Reporting of Observational studies in Epidemiology (STROBE) and the 2015 Standards for Reporting Diagnostic Accuracy Studies (STARD) guidelines. Checklists and additional details are given in Additional file 1, Supplementary Tables 3 and 4 respectively.

## Results

### General population characteristics and outcome

Of 1939 enrolled participants, 1922 (99.1%) had sufficient diagnostic data for analysis, including 667 (34.7%) in Sudan, 300 (15.6%) in DRC, 577 (30.0%) in Nepal, and 378 (19.7%) in Cambodia. Baseline characteristics, pre-inclusion pathway, and clinical outcomes of study participants (*n* = 1922) are presented in Table 1 for the whole cohort and per country. The median age of the overall study population was 35 years (range: 5–99 years), but younger in DRC (median: 19 years) and older in Cambodia (median: 47 years). The male-to-female ratio was 0.94, with only minor differences between countries. The median duration of fever was 14 days (interquartile range [IQR]: 9–28 days) and similar in all sites. A large proportion of the patients with fever also had respiratory (1325, 69%), digestive (1145, 60%), or genitourinary symptoms (710, 37%). This latter presentation was particularly frequent in Sudan (505/668, 76%). An important proportion of participants in Cambodia had co-morbidities (196/378, 52%), including about 10% with HIV infection. About 25% of patients in total had been exposed to antibiotics before inclusion, with higher proportions in Nepal (35.9%).

**Table 1:** Baseline characteristics and outcomes of patients (*n* = 1922) evaluated for persistent fever, per study country

	Sudan *n* = 667	DR Congo *n* = 300	Nepal *n* = 577	Cambodia *n* = 378	Total *n* = 1922
Epidemiological data					
Age in year, median (Q1–Q3)	35 (20–48)	19 (7–40)	33 (20–50)	47 (35–58)	35 (20–50)
Age category					
5–17 years	132 (19.8)	137 (45.7)	107 (18.5)	–	376 (19.6)
18–49 years	379 (56.8)	124 (41.3)	308 (53.4)	210 (55.6)	1021 (53.1)
≥ 50 years	156 (23.4)	39 (13.0)	162 (28.1)	168 (44.4)	525 (27.3)
Female gender	382 (57.3)	164 (54.7)	251 (43.5)	193 (51.1)	990 (51.5)
Clinical features					
Duration of fever before inclusion in days, median (Q1–Q3)	15 (10–28)	14 (7–28)	14 (8–28)	14 (10–35)	14 (9–28)
Reported antibiotic exposure^a^	168 (25.2)	29 (9.7)	205 (35.5)	24 (6.5)	426 (22.2)
Reported antimalarial exposure^a^	181 (27.1)	13 (4.3)	26 (4.5)	4 (1.1)	224 (11.7)
Underlying co-morbidities	51 (7.6)	31 (9.4)	44 (7.7)	196 (51.8)	322 (16.7)
*HIV infection*	*5 (0.7)*	*5 (1.7)*	*5 (0.9)*	*41 (10.8)*	*56 (2.9)*
*Diabetes mellitus*	*12 (1.8)*	*4 (1.3)*	*14 (2.4)*	*44 (11.6)*	*74 (3.9)*
*Arterial hypertension/heart disease*	*23 (3.4)*	*16 (5.3)*	*16 (2.8)*	*50 (13.2)*	*105 (5.5)*
*Chronic viral hepatitis/liver cirrhosis*	–	–	–	*27 (7.1)*	*27 (1.4)*
*Rheumatic disorders*	*2 (0.3)*	–	*2 (0.3)*	*17 (4.5)*	*21 (1.1)*
*Epilepsy/neurological disorders*	–	*6 (2.0)*	*2 (0.3)*	*5 (1.3)*	*13 (0.7)*
Associated symptoms					
Respiratory	518 (77.7)	136 (45.3)	352 (61.0)	319 (84.4)	1325 (68.9)
Digestive	530 (79.5)	181 (60.3)	207 (35.9)	227 (60.1)	1145 (59.6)
Cutaneous	165 (24.7)	39 (13)	43 (7.5)	89 (23.5)	336 (17.5)
Genitourinary	505 (75.7)	30 (10.0)	81 (14.0)	94 (24.9)	710 (36.9)
Treatment and outcome					
Immediate hospital admission	72 (10.8)	209 (69.7)	216 (37.4)	207 (54.8)	704 (36.6)
Duration of hospital stay in days, median (Q1–Q3)	16 (10–17)	6 (4–10)	5 (3–10)	6 (3–10)	6 (3–12)
Antibiotics prescribed after inclusion	536 (79.2)	166 (53.3)	312 (54.1)	307 (81.2)	1321 (68.7)
Antimalarials prescribed after inclusion	41 (6.1)	138 (46.0)	39 (6.8)	27 (7.1)	245 (12.7)
Lost to follow-up or missing information	237 (35.8)	95 (31.7)	70 (12.0)	71 (18.8)	475 (24.7)
Reported deaths	0 (0)	4 (1.3)	17 (2.9)	42 (11.1)	63 (3.3)
Resolved or improving	301 (45.1)	182 (60.7)	482 (83.5)	232 (61.4)	1197 (62.3)
No improvement or sequelae	127 (19.0)	19 (6.3)	8 (1.4)	33 (7.9)	187 (9.7)

All results are presented as *n* (%) except mentioned otherwise

DR Congo denotes Democratic Republic of Congo; Q1–Q3 interquartile range

^a^Up to 1 month before inclusion

In total, 704 (36.6%) participants were immediately admitted to the hospital, in particular in Cambodia and DRC. At inclusion, antibiotics were prescribed in more than 70% of patients with persistent fever, except in DRC where about half received antimalarials. For 475 (24.7%) participants, data on final assessment (at 12 weeks post-inclusion) were incomplete. In almost all these cases, data on the 4-week follow-up visit were also missing. Sixty-three patients died, corresponding to 3.3% of the whole cohort or to 4.4% (63/1447) of those participants for whom outcome data were complete. This proportion was particularly high in Cambodia (42/305, 13.8%). Another 187 patients (9.7%) did not report any significant improvement or suffered some sequelae at the final assessment.

### Etiological spectrum and frequency of priority infections

Table 2 shows the frequency of the 12 priority conditions and other clinical diagnoses for the 1922 participants and per study country. Eighty-one patients (4.2%) had more than one confirmed diagnosis that could explain the fever. A total of 452 patients (23.5%) were diagnosed with at least one of the six ubiquitous infections on which this study focuses: malaria in 154 (8.0%) patients; tuberculosis in 129 (6.7%), including 86 (67%) with pulmonary tuberculosis; leptospirosis in 77 (4.0%); rickettsiosis in 44 (2.3%), mainly murine typhus (due to *Rickettsia typhi*) and to a lesser extent scrub typhus (*Orientia tsutsugamushi*) and spotted fever (*Rickettsia* spp.); enteric fever in 34 (1.8%), with a predominance of *Salmonella* Typhi (23/34, 67.6%); and new HIV diagnosis in 14 (0.7%). For three of the ubiquitous priority infections, there were clear differences in frequency across the sites: malaria was much more frequent in African than in Asian sites; tuberculosis was particularly frequent in Cambodia; and rickettsiosis was frequent in Nepal and infrequent in Sudan. The frequencies of the remaining three ubiquitous infections were similar across the sites. Seven patients with tuberculosis died (two in DRC, one in Nepal and four in Cambodia). One patient with enteric fever and seven with leptospirosis died, all in Cambodia. Two fatalities were reported in patients newly diagnosis with HIV, both in Nepal.

**Table 2:** Frequency of NIDIAG priority infections and of other clinical diagnoses in 1922 patients evaluated for persistent fever, per study country

	Sudan *n* = 667	DR Congo *n* = 300	Nepal *n* = 577	Cambodia *n* = 378	Total *n* = 1922
NIDIAG priority conditions					
Enteric fever	11 (1.6)	9 (3.0)	8 (1.4)^a^	6 (1.6)	34 (1.8)
*Salmonella* Typhi	9	9	5	–	23
*Salmonella* Paratyphi A	2	–	3	6	11
Leptospirosis	13 (1.8)	10 (3.3)	30 (5.2)	24 (6.3)	77 (4.0)
Confirmed	1	5	13	12	31
Probable	12	5	17	12	46
Rickettsiosis	1 (0.1)	5 (1.7)	28 (4.7)	10 (2.6)	44 (2.3)
*Rickettsia typhi*^*b*^	–	5	5	8	18
*Orientia tsutsugamushi*^*c*^	–	–	11	2	13
*Rickettsia spp.*^*d*^	1	–	12	–	13
Relapsing fever	7 (1.0)	–	4 (0.7)	1 (0.3)	12 (0.6)
Brucellosis	28 (4.2)	–	–	–	28 (1.5)
Melioidosis	–	–	–	16 (4.2)	16 (0.8)
Visceral leishmaniasis	65 (9.7)	–	56 (9.7)	–	119 (6.2)
Human African trypanosomiasis	–	2 (0.7)	–	–	2 (0.1)
Amebic liver abscess	–	–	1 (0.2)	11 (2.9)	12 (0.6)
Malaria	55 (8.2)	96 (32.0)	–	4 (1.1)	154 (8.0)
Tuberculosis^e^	8 (1.2)	18 (6.0)	27 (4.7)	76 (20.1)	129 (6.7)
New HIV diagnosis/opportunistic infection (other than tuberculosis)	4 (0.6)	4 (1.3)	4 (0.7)	2 (0.5)	14 (0.7)
Other clinical diagnoses					
Other (suspected) systemic bacterial infections	2 (0.3)	11 (3.7)	2 (0.3)	29 (7.7)	44 (2.3)
Suspected focal bacterial infections	101 (5.3)	47 (15.7)	55 (9.5)	161 (42.6)	364 (18.9)
Pneumonia	23 (3.4)	22 (7.3)	20 (3.5)	83 (22.0)	148 (7.7)
Abdominal/intestinal infection	12 (1.8)	7 (2.3)	4 (0.7)	26 (6.9)	49 (2.5)
Genitourinary infection	63 (9.4)	13 (4.3)	31 (5.4)	26 (6.9)	133 (6.5)
Skin and soft tissue infection	3 (0.4)	5 (1.7)	–	26 (6.9)	34 (1.4)
Suspected viral infection (respiratory/other)	54 (8.1)	29 (9.7)	76 (13.2)	5 (1.3)	164 (8.5)
Other infections (parasitic, fungal)	2 (0.3)	2 (0.7)	–	6 (1.6)	10 (0.5)
Non-infectious etiologies	9 (1.3)	1 (0.3)	11 (1.9)	17 (4.5)	38 (2.0)
Unknown/unspecified cause	333 (49.9)	80 (26.7)	278 (48.2)	54 (14.3)	745 (38.8)

All results are presented as *n* (%) except when mentioned otherwise

Enteric fever was diagnosed together with another infection in 8 cases (brucellosis, *n* = 3; visceral leishmaniasis, *n* = 2; malaria, *n* = 2; rickettsiosis, *n* = 2; HIV, *n* = 1). Leptospirosis was diagnosed together with another infection in 13 cases (tuberculosis, *n* = 3; malaria, *n* = 2; pneumonia, *n* = 2; skin/soft tissue infection, *n* = 2; visceral leishmaniasis, *n* = 1; others, *n* = 3). Rickettsiosis was diagnosed together with another infection in 6 cases (visceral leishmaniasis, *n* = 4; enteric fever, *n* = 2)

^a^ One case of enteric fever was due to *Salmonella spp.* (in Nepal)*.*

^b^ Confirmed in 12 cases, either by PCR (*n* = 3) or seroconversion (*n* = 9); probable in 6 cases

^c^ Confirmed in 4 cases either by PCR (*n* = 1) or seroconversion (*n* = 3); probable in 9 cases

^d^ Confirmed in 13 cases by PCR

^e^ Including 86 (67%) pulmonary and 43 (33%) extrapulmonary tuberculosis

Regarding the other NIDIAG priority conditions, visceral leishmaniasis was frequently (and exclusively) diagnosed in Nepal and Sudan. Three conditions were diagnosed in one country only: melioidosis in Cambodia, brucellosis in Sudan, and HAT in DRC. Amebic liver abscess and relapsing fever were infrequent.

Other common causes of persistent fever included (suspected) systemic (*n* = 44, 2.3%) and focal (*n* = 364, 18.9%) bacterial infections (mainly pneumonia and genitourinary infection). Viral infections were suspected in less than 10% of all cases. A large proportion of etiologies remained unknown (745, 38.8%), despite an extensive standardized evaluation.

### Clinical and laboratory presentation of ubiquitous priority infections

Table 3 shows the frequency of symptoms, signs and laboratory features at presentation per ubiquitous infection (as single diagnosis), compared with all other pooled etiologies. The following features (with their frequencies and LR+) were observed in one of the six infections more often than in the rest of the cohort: in malaria cases, abdominal pain (66%; LR+ 1.5) and anemia, defined as hemoglobin level < 10 mg/dL (43%; LR+ 1.8); in tuberculosis, cough (92%; LR+ 2.0), cachexia, defined as body mass index < 18 (38%; LR+ 2.7), and anemia (36%; LR 1.5); in leptospirosis patients, white blood cell count > 10,000/μL (30%; LR+ 1.2) and elevated alanine aminotransferase (ALT) level (34%; LR+ 1.5); in enteric fever cases, abdominal pain (58%; LR+ 1.3), diarrhea (42%; LR+ 3.3), vomiting (31%; LR+ 1.7), and elevated ALT level (72%; LR+ 3.3); in patients with rickettsiosis, vomiting (24%; LR+ 1.3) and elevated ALT level (68%; LR+ 3.1); in newly diagnosed HIV patients, cachexia (42%; LR+ 2.7), and anemia (67%; LR+ 2.7). Of all investigated features, only the presence of hyperleukocytosis had a good excluding power (LR- 0.16) for enteric fever.

**Table 3:** Frequency of clinical and basic laboratory features in study patients with a single diagnosis of ubiquitous priority infection

Features	Malaria (*n* = 131)	Tuberculosis (*n* = 120)	Enteric fever (*n* = 26)	Leptospirosis (*n* = 64)	Rickettsiosis (*n* = 38)	New HIV (*n* = 12)	Other diagnoses (*n* = 1531)
**Symptoms**							
Headache	92 (70 ; 62–78)	66 (55 ; 46–64)	18 (69; 50–84)	44 (69; 57–79)	27 (71; 55–83)	5 (42; 19–68)	1002/1527 (66; 63–68)
Myalgia	29 (22; 16–30)	13 (11; 6–18)	3 (12; 4–29)	17 (27; 17–38)	9 (24; 13–39)	4 (33; 14–61)	509/1528 (33; 31–36)
Cough	32 (24; 18–32)	110 (92; 85–95)	9 (35; 19–54)	31 (48; 37–60)	15 (39; 26–55)	6 (50; 25–75)	750/1530 (49; 47–52)
Dyspnea	17 (13; 8–20)	68 (57; 43–65)	3 (12; 4–29)	17 (27; 17–38)	4/37 (11; 4–25)	3 (25; 22–27)	374/1521 (25; 22–27)
Vomiting	40 (31; 23–39)	14 (12; 7–19)	8 (31; 16–50)	12 (19; 11–30)	9 (24; 13–39)	1 (8; 1–35)	276/1530 (18; 16–20)
Diarrhea	5 (4; 2–9)	19 (16; 10–23)	11 (42; 26–61)	6 (9; 4–19)	3 (8; 3–21)	3 (25; 9–53)	231/1791 (13; 11–15)
Abdominal pain	86 (66; 57–73)	35 (29; 22–38)	15 (58; 39–74)	20 (31; 21–43)	10 (26; 15–42)	4 (33; 14–61)	681/1529 (45; 42–47)
Weight loss	60 (46; 38–55)	104/114 (91; 85–95)	14 (54; 35–71)	34/61 (56; 43–67)	14/27 (52; 34–69)	9 (75; 47–91)	857/1469 (58; 56–61)
**Signs**							
Skin lesion or rash	14 (11; 6–17)	11 (9; 5–16)	0 (0; 0–13)	7 (11; 5–21)	0 (0; 0–9)	0 (0; 0–24)	132/1530 (9; 7–10)
Lymphadenopathies	6 (5; 2–10)	3 (3; 1–7)	0 (0; 0–13)	1 (1.5; 0–8)	0 (0; 0–9)	2 (17; 5–45)	71 (5; 4–6)
Abdominal tenderness	36 (28; 21–36)	29 (24; 17–33)	10 (38; 22–57)	17 (27; 17–38)	5 (13; 6–27)	4 (33; 14–61)	479 (31; 24–34)
Hepatomegaly	12 (9; 5–15)	5 (4; 2–9)	2 (8; 2–24)	2 (3; 1–11)	6 (16; 7–30)	0 (0; 0–24)	144 (9; 8–11)
Splenomegaly	22 (17; 11–24)	4 (3; 1–8)	3 (12; 4–29)	4 (6; 2–15)	5 (13; 6–27)	0 (0; 0–24)	152 (10; 9–12)
Jaundice	9 (7; 4–13)	2 (2; 0–6)	1 (4; 1–19)	4 (6; 2–15)	2 (5; 1–17)	0 (0; 0–24)	73 (5; 4–6)
Cachexia	26 (20; 14–27)	45 (38; 29–46)	3 (12; 4–29)	6 (9; 4–19)	4 (11; 4–24)	5 (42; 19–68)	212/1530 (14; 12–16)
**Laboratory results**							
Hemoglobin level < 10 g/dL)	56 (43; 35–51)	43 (36; 28–45)	5 (19; 9–38)	11 (17; 10–28)	7/37 (19; 9–34)	8 (67; 39–86)	342/1522 (23; 21–25)
WBC count > 10,000/μL	15/106 (14; 9–22)	54/117 (46; 37 – 55)	1/23 (4; 1–21)	21/63 (33; 23–46)	10/36 (28; 16–44)	2 (17; 5–45)	377/1482 (25; 23–28)
Elevated alanine aminotransferase	15/130 (12; 7–18)	38 (32; 24–40)	18/25 (72; 52–89)	22 (34; 24–47)	23/34 (68; 51–81)	1 (8; 1–35)	313/1494 (21; 19–23)
Raised creatinine level	2/88 (2; 1–8)	15/113 (13; 8–21)	3/18 (17; 6–39)	4/53 (8; 3–18)	5/35 (14; 6–29)	1/8 (12; 2–47)	94/902 (10; 9–13)
Protein (urine strip)	40/128 (31; 24–40)	24/110 (22; 15–30)	12 (46; 29–65)	7/61 (11; 6–22)	5 (13; 6–27)	4 (33; 14–61)	330/1494 (22; 20–24)

All results are *n* or *n*/*n* (%; 95% confidence interval)

### Performance of RDTs for enteric fever/typhoid fever and leptospirosis

Table 4 shows the sensitivity, specificity, LRs, and post-test probabilities for the three index RDTs targeting typhoid fever. Taking all single diagnoses of enteric fever together (*n* = 26), the sensitivity of Test-it^TM^ Typhoid IgM Lateral Flow Assay and Typhidot Rapid IgM was about 50%. When we restricted the analysis only to *S.* Typhi infections (typhoid fever cases), for which theses RDTs have been designed, the sensitivity increased up to approximately 70%. However, since the specificity was suboptimal (< 90% for both tests), each test displayed a LR+ of about 5 for both enteric fever and *S.* Typhi infection. The SD Bioline Salmonella typhi IgG/IgM had different performance characteristics: the sensitivity was lower than that of the other two RDTS, but the specificity was higher, resulting in a LR+ of 15. This could increase the probability of both enteric fever and *S*. Typhi infection from < 2% at baseline to 20% if positive. The sensitivity of both RDTs targeting leptospirosis (Table 5) was very low (< 20%), resulting in weak confirming power (LR+ = 3) despite high specificities (> 90%).

**Table 4:** Diagnostic performance of the study RDTs for all pooled cases of enteric fever and for *Salmonella* Typhi infections only

	SD Bioline Salmonella typhi IgG/IgM	Test-it^TM^ Typhoid IgM Lateral Flow Assay	Typhidot Rapid IgM
Enteric fever			
Sensitivity	5/26 (19; 9–38)	8/15 (53; 30–75)	12/26 (46; 29–65)
Specificity	1867/1892 (98.7; 97.7–99.7)	689/771 (89.4; 87.0–91.0)	1605/1862 (86.2; 84.7–87.6)
Positive likelihood ratio	14.6	5.0	3.3
Negative likelihood ratio	0.82	0.52	0.62
Post-test probability if positive test (pre-test = 1.8%)^a^	21.1%	8.4%	5.8%
Post-test probability if negative test (pre-test = 1.8%)^a^	1.5%	0.9%	1.1%
*Salmonella* Typhi infection			
Sensitivity	5/17 (29; 13–53)	6/9 (67; 35–88)	10/17 (59; 36–78)
Specificity	1876/1901 (98.7; 97.7–99.7)	693/777 (89.2; 86.6–91.5)	1612/1871 (86.2; 84.7–87.6)
Positive likelihood ratio	22.4	6.2	4.2
Negative likelihood ratio	0.70	0.37	0.48
Post-test probability of if positive test (pre-test = 1.2%)^a^	21.4%	7.0%	4.9%
Post-test probability if negative test (pre-test = 1.2%)^a^	0.9%	0.5%	0.6%

Sensitivity and specificity are reported as *n*/*n* (%; 95% confidence interval)

^a^ Pre-test probabilities indicate the frequency of enteric fever (i.e., 34/1922, 1.8%) and *S.* Typhi infection (i.e., 23/1922, 1.2%) in the study cohort

**Table 5:** Diagnostic performance of the study RDTs for all pooled cases of leptospirosis

	SD Bioline Leptospira IgG/IgM	Test-it^TM^ Leptospira IgM Lateral Flow Assay
Sensitivity	6/64 (9; 4–19)	5/31 (16; 7–33)
Specificity	1804/1853 (97.4; 96.5–98.4)	785/819 (95.8; 94.3–98.2)
Positive likelihood ratio	3.5	3.8
Negative likelihood ratio	0.93	0.88
Post-test probability if positive test (pre-test = 4.0%)^a^	12.6%	13.7%
Post-test probability if negative test (pre-test = 4.0%)^a^	3.7%	3.5%

Sensitivity and specificity are reported as *n*/*n* (%; 95% confidence interval)

^a^Pre-test probability indicates the frequency of leptospirosis in the study cohort (i.e., 4.0%)

## Discussion

The epidemiology of persistent fever in the tropics has been virtually unexplored so far, despite the fact that this clinical entity is particularly challenging to manage. In this multicentric study that systematically investigated a pre-established set of severe and treatable infections, 23.5% of all cases were diagnosed with one of the six ubiquitous priority infections, including 8.1% with either leptospirosis, rickettsiosis, or enteric fever, beside the “more expected” malaria, tuberculosis, and HIV infection. In the heterogeneous mix of etiologies of this particular scenario, our analysis did not yield strong clinical or basic laboratory features that could reliably distinguish any of them. Moreover, our evaluation of antibody-based immunochromatographic RDTs targeting typhoid fever and leptospirosis highlighted the current vacuum of acceptably accurate diagnostic tests for these conditions in low-resource settings.

The NIDIAG study on persistent fever had several strengths. We deployed extensive, systematic, and quality-assured testing at the point of care in varied and/or remote settings and included a large cohort of patients. Reference standard testing was pre-specified by experts and uniformly applied. Recruitment was conducted in several tropical countries and in varied settings, including outpatient clinics and referral hospitals, making our findings broadly applicable. The study population was also heterogeneous in age, gender, and presence of comorbidity, with no particular group specifically excluded (except children < 5 years), somehow reflecting the complex nature of the real life clinical practice. Finally, the multicentric design allowed a more robust search for predictors by grouping all ubiquitous priority infections that were found at rather low frequency in each study site.

However, the present study must be interpreted in its context. First, while the approach of the targeted infections was robust and standardized between study sites, diagnostic platforms for all other diseases were basic, and biochemistry and hematology were not harmonized. This left much uncertainty regarding the diagnosis of all other conditions, and this resulted in a large proportion of unknown etiologies, in fact rather similar to what is observed in most AFI studies. Second, the methods to diagnose some conditions under study may be considered as imperfect reference standards, since they all have notorious limitations both in sensitivity and clinical specificity. For example, sensitivity of a single blood culture for diagnosing enteric fever is 60% at best, tends to decrease in the second week of fever, and is impaired by any prior empiric antibiotic treatment [15]. PCR assays targeting leptospirosis in serum and/or urine have an overall sensitivity of 70%, varying in the course of the infection and possibly lower in the second week of the illness [16]. Detection of rickettsial DNA by PCR in blood/serum is transient and sensitivity may be as low as 18% for murine typhus [17]. Paired serology on acute and convalescent sera could not be performed in about 25% of the cases who did not come back for follow-up. Besides this possibility of false-negative results, there is also an issue of false-positive results. Indeed, attributing a fever episode to the presence of a pathogen is not always appropriate, as is well known for the presence of malaria parasites in blood smears in hyperendemic areas like the DRC. Similarly, a few case-control studies investigating the etiologies of AFI have demonstrated that some individuals may be carriers of *Leptospira* or *Rickettsia* spp*.* pathogens (as detected by PCR) without presenting any symptom [18]. The absence of controls in our study did not allow to fully apprehend the status of infection versus disease, despite strict case ascertainment. Finally, the substantial mortality rate of persistent fever is likely underestimated since a sizeable proportion of participants were lost to follow-up and the most severe cases at presentation were not included in this study.

It is not possible to compare our data with other studies on persistent fever, which are virtually absent. As expected, tuberculosis and HIV diagnoses were more frequent than in AFI studies. Furthermore, one should consider that malaria can be also a rather frequent etiology of persistent fever, in particular in holoendemic areas. Here, enteric fever, leptospirosis, and rickettsiosis were less frequent (8%) than in AFI studies, where these three infections together were found in 10% up to 30% of the cases, particularly in Asia [19, 20]. However, it remains important to promptly recognize each of these conditions, at whatever timing of presentation, since morbidity and mortality are reported as substantial in more advanced stages [21–26] and treatment is specific [18, 27, 28].

The frequencies of clinical and laboratory features for the six ubiquitous infections on which this study focuses were rather similar to those observed in other case series, with no apparent impact of the large and variable period between disease onset and inclusion. Very few of them were discriminative however in the clinical scenario under study, with moderate confirming power at best (LR+ of about 3) [29], underlining that disease presentation of many infections may remain undifferentiated also in later stages (more advanced or in partial recovery). Of note, we did not find skin lesions in cases of rickettsiosis, notably in patients with scrub typhus where the presence of an eschar is usually considered as a key feature [18, 19]. Reasons for the discrepancy with AFI studies likely reside in the late inclusion after symptom onset, at a moment an eschar is resolving or exposure to specific vectors tends to be overlooked. Other possible explanations are that eschar frequency may vary across geographical areas and that in this series, murine typhus was predominant. In fact, only an elevated level of ALT was a moderate predictor of enteric fever [30] and rickettsiosis [19], but the usefulness of this feature in clinical practice is questionable.

In general, the sensitivities of antibody-based lateral-flow RDTs were disappointing for typhoid fever and leptospirosis. Also, in view of low prevalence, the specificities of some RDTs also need considerable improvement. Meaningful comparisons with other diagnostic studies are difficult because few were well-designed and sufficiently large, making reported accuracy data uncertain and variable. Also, the timing of evaluation after fever onset was unusually delayed in our study, although one would have expected the sensitivity of antibody-based tests to be higher after the first week of symptoms. The contribution of *Salmonella* Paratyphi A in the caseload of enteric fever may partly explain the suboptimal performance of the studied RDTs compared to published data [31], although performance did not improve that much when restricting to *Salmonella* Typhi cases. For leptospirosis, reasons for the surprisingly low accuracy of antibody-based lateral-flow assays are unclear, when compared to the limited literature, where overall sensitivities from 50 to 90% are reported [32]. However, systematic reviews suggest that diagnostic evaluations vary substantially, in particular regarding the imperfect reference standards that could be prone to misclassifications in almost all studies [32]. All in all, our observations suggest that design and engineering are still needed to improve the specific diagnosis of some of the studied conditions [14].

In addition to the 23.5% of 1922 patients diagnosed with at least one of the ubiquitous priority infections, a striking finding among our cohort of patients with persistent fever was the high frequency of suspected but unconfirmed systemic or focal bacterial infections (overall 21.2%; Table 2). This observation underlines the need of evaluating the clinical usefulness of generic point-of-care bacterial biomarkers also in the persistent fever syndrome [33]. However, without current diagnostic alternatives to bacterial cultures for the diagnosis of enteric fever and susceptibility testing in general, this finding also emphasizes the relevance of prioritizing implementation of clinical bacteriology in low-resource settings, as recommended for secondary-level hospitals in the WHO Model Essential Diagnostics List. Tailored bacteriology laboratory interventions have been shown to be feasible [34] and have substantial benefits beyond diagnostics targeting single diseases [35].

Finally, it is worth highlighting that besides the ubiquitous infections reported here, this study demonstrates the geographical importance of some parasitic (VL) and bacterial (brucellosis, melioidosis) diseases in the persistent fever syndrome. These observations will be reported elsewhere.

## Conclusions

This multicentric study provides important and innovative insights into the frequency of the main and ubiquitous conditions causing persistent fever in the tropics, a neglected clinical scenario. It also highlights the unsolved challenges in diagnosing important diseases such as leptospirosis, rickettsiosis, and enteric fever. In addition to increasing clinical awareness about these etiologies, our observations underline the urgent need of further developing both disease-specific and generic field diagnostics to optimize their case management.

## Supplementary Information

**Additional file 1: Table S1.** Diagnostic ascertainment of the 12 (NIDIAG) target priority infections; **Table S2**. Case ascertainment of conditions not targeted by the NIDIAG workup; **Table S3.** STROBE Checklist of items for cross-sectional studies (NIDIAG); **Table S4.** STARD checklist NIDIAG study.

## Data Availability

The datasets used and/or analyzed during the current study are available from the senior author on reasonable request (ebottieau@itg.be) and will be submitted for approval by the NIDIAG consortium.

## Abbreviations

AFI: Acute febrile illness
FIEBRE: Febrile Illness Evaluation in a Broad Range of Endemicities
NIDIAG: Better Diagnosis of Neglected Infectious Diseases
VL: Visceral leishmaniasis
HAT: Human African trypanosomiasis
RDT: Rapid diagnostic test
DRC: Democratic Republic of Congo
HIV: Human immunodeficiency virus
ALT: Alanine aminotransferase
