# Supplementary material for: Etiological spectrum of persistent fever in the tropics and predictors of ubiquitous infections: a prospective four-country study with pooled analysis
Source: BMC Med. 2022 May 2;20:144. doi: 10.1186/s12916-022-02347-8 (PMC9059373; doi:10.1186/s12916-022-02347-8)
Supplement: Supplementary file 1 — Additional file 1: Table S1. Diagnostic ascertainment of the 12 (NIDIAG) target priority infections; Table S2. Case ascertainment of conditions not targeted by the NIDIAG workup; Table S3. STROBE Checklist of items for cross-sectional studies (NIDIAG); Table S4. STARD checklist NIDIAG study. [file 12916_2022_2347_MOESM1_ESM.docx]

# **Supplementary information**

Additional file 1: A .pdf file providing the Supplementary Tables: **Table S1** : Diagnostic ascertainment of the 12 (NIDIAG) target priority infections; **Table S2**: Case ascertainment of conditions not targeted by the NIDIAG workup; **Table S3**: STROBE Checklist of items for cross-sectional studies (NIDIAG); **Table S4**: STARD checklist NIDIAG study

**Table S1:** Diagnostic ascertainment of the 12 (NIDIAG) target priority infections

| **Target infection** | **Case definition** |
| --- | --- |
| Enteric (typhoid, paratyphoid) fever | **Confirmed:** Isolation of *Salmonella* Typhi (typhoid fever) or *Salmonella* Paratyphi A or B (paratyphoid fever) in blood or bone marrow cultures |
| Leptospirosis | **Confirmed:** (a) Positive PCR on serum or urine **and/or**  (b) Seroconversion or ≥4-fold increase in specific antibody titers, as measured by microagglutination test (MAT), between initial and follow-up sera  **Probable:** Positive specific serology on a single serum sample (MAT) |
| Rickettsial infection | **Confirmed:** (a) Positive PCR on serum **and/or**  (b) Seroconversion or ≥4-fold increase in specific IgM titers by immunofluorescence assay (IFA) between initial and follow-up sera  **Probable:** Positive serology (IgM on IFA) on single serum sample |
| Relapsing fever | **Confirmed:** (a) Microscopic observation of spirochetes in Giemsa-stained thin or thick blood smears **and/or** (b) Positive PCR for *Borrelia spp.* on serum |
|  |  |
| Brucellosis | **Confirmed:** (a) Isolation of *Brucella* spp.^d^ in prolonged cultures of blood or bone marrow **and/or**  (b) Seroconversion or 4-fold increase in antibody titers, as determined with the Rose Bengal agglutination test (RBT) and confirmed by serum agglutination test (SAT) or competitive ELISA (cELISA)  **Probable:** High single RBT titer (≥1:320) confirmed by SAT or cELISA |
| Melioidosis | **Confirmed:** Isolation of *Burkholderia pseudomallei* from culture of blood or other body sites (e.g. throat, urine, joint fluid, soft tissue) |
| Visceral leishmaniasis | **Confirmed:** (a) Microscopic observation of *Leishmania donovani* bodies from samples obtained by lymph node puncture or bone marrow aspirate **and/or**  (b) Microscopic observation of *Leishmania* promastigotes in culture media inoculated with biological fluid obtained by lymph node puncture or bone marrow aspiration **and/or**  (c) Positive direct agglutination test (DAT) result (titer≥ 1:6400 in Nepal and ≥1:3200 in Sudan) and good clinical response^b^ to anti-leishmanial treatment and no history of previous VL  **Probable:** Positive DAT result (titer≥ 1:6400 in Nepal and ≥1:3200 in  Sudan) **and** assessment of clinical response not done or not possible |
| Human African trypanosomiasis | **Confirmed:** Microscopic demonstration of trypanosomes in (i) cervical lymph node fluid obtained by puncture, (ii) blood using mini-anion exchange centrifugation technique (mAECT), and/or (iii) CSF using modified single centrifugation  **Probable:** (a) Positive CATT titre ≥ 1/16, (b) presence of symptoms/signs consistent with HAT, (c) absence of trypanosomes in blood, LN or CSF, and (d) anti-trypanosomal treatment initiated by the treating physician |
| Amebic liver abscess | **Confirmed:** liver ultrasound or abdominal CT scan showing ≥1 lesions consistent with liver abscess with positive serology by immunofluorescence assay (IFA) **and** clinical response to specific treatment **Probable:** US or CT showing ≥1 lesions consistent with liver abscess with positive serology (IFA) **and** no assessment of clinical response to specific treatment |
| Malaria | **Confirmed:** Positive microscopic examination (asexual parasites) of thin or thick blood film **and/or** positive malaria Ag Pf/pan pLDH RDT, Standard Diagnostics, Korea |
| Tuberculosis | **Confirmed:** (a) Microscopic observation of acid fast bacilli **and/or**  (b) positive culture **and/or**  (c) positive PCR for *Mycobacterium tuberculosis*  on sputum or other biological fluid (urine, pus, ascites or pleural fluid, cerebrospinal fluid)  **Probable (pulmonary TB):** Cough for ≥2 weeks **and** at least one of the following: (a) bloody sputum; (b) suggestive chest x-ray; (c) weight loss and night sweats; (d) no improvement with regular antibiotics |
| HIV | **Confirmed:** (a) Three positive RDTs, according to the national program strategy), including Uni-Gold HIV-1/2 (Trinity Biotech, Ireland); Determine (Inverness Medical, Japan); Gold Colloidal (Shanghai Kehua Bio-engineering, China) **or**  (b) Two positive RDTs and one positive ELISA |

**Table S2:** Case ascertainment of conditions not targeted by the NIDIAG workup

| **Other conditions** | **Case definition** |
| --- | --- |
| Other (suspected) systemic bacterial infections | **Bacteremia**: includes all cases with bacteria isolated in blood cultures, excluding those considered contaminants; and excluding *Salmonella*, *Brucella* and *Burkholderia* species.  **Bacterial meningo-encephalitis**  Confirmed: Clinically compatible case with identification of a bacterial species from either CSF (by direct microscopy or by culture) or blood culture; OR  Probable: In case CSF culture was negative or not performed: a CSF white blood cell count of >1000/µL, CSF protein >200 mg/dL, and/or glucose <40 mg/dL (with a CSF/serum glucose ratio of ≤0.4), and tuberculous meningitis not suspected or confirmed  **Clinical sepsis**: includes patients with clinical presentation of sepsis or septic shock, based on clinical estimation of the attending research physician; and absence of another defined specific diagnosis (sepsis without apparent focus) |
| Pneumonia | Confirmed: Clinical suspicion and lung infiltrate on chest X-rays  Probable: Clinical suspicion only: fever, cough AND presence of crackles on lung auscultation |
| Abdominal/intestinal infection | Cases with clinically high suspicion of bacterial enteritis/dysentery (blood stained stool, high frequency of bouts of stool) with or without presence of pus cells/red blood cells in stool sample (stool culture not available)  Cases with high clinical certainty of bacterial intra-abdominal infection, with confirmation by surgical exploration and/or by ultrasound confirmation and/or by ascitic fluid analysis for the following diagnoses: cholangitis, cholecystitis, peritonitis, spontaneous bacterial peritonitis, bacterial liver abscess |
| Genitourinary infection | **Urinary tract infection:**  Confirmed: Clinical suspicion and positive bacterial culture  Probable: Clinical suspicion (symptom(s): pain in passing urine, increased frequency, loin pain; signs: suprapubic and/or loin pain on palpation) and positive urine dipstick (positive nitrite and/or leukocytes ≥75/μl or Leukocyte esterase ++/+++)  **Pelvic inflammatory disease (PID):**  Confirmed: Clinical suspicion and positive bacterial culture  Probable: Clinical suspicion and positive PCR/culture of genital secretion for *C. Trachomatis*, *N. gonorrhoeae* or other bacteria associated with PID |
| Skin and soft tissue infection | Clinical diagnosis of cellulitis, erysipelas, skin abscess, muscle abscess, infected eschar, adenitis, mastitis |
| (Suspected) viral infection (respiratory or other) | **Respiratory tract infection (RTI):**  Probable: Clinical suspicion only: dry or productive cough, runny nose, sore throat, sinus or hear pain after exclusion of pneumonia, tuberculosis or other systemic condition (e.g. enteric fever)  **Meningo-encephalitis**: Cases with clinical features of meningoencephalitis and pleocytosis at CSF examination (>5 WBC/µL) but without demonstration of etiological pathogen and none of the above mentioned criteria for bacterial meningitis were fulfilled |
| Other infections (parasitic, fungal) | **Confirmed:** only if documented by parasite examination, fungal culture or histology |

**Table S3:** STROBE Checklist of items for *cross-sectional studies* (NIDIAG)

|  | Item No | Recommendation | Page No |
| --- | --- | --- | --- |
| **Title and abstract** | 1 | (*a*) Indicate the study’s design with a commonly used term in the title or the abstract | 1 |
|  |  | (*b*) Provide in the abstract an informative and balanced summary of what was done and what was found | 2-3 |
| Introduction | | | |
| Background/rationale | 2 | Explain the scientific background and rationale for the investigation being reported | 4-5 |
| Objectives | 3 | State specific objectives, including any prespecified hypotheses | 5 (lines 97-102) |
| Methods | | | |
| Study design | 4 | Present key elements of study design early in the paper | 5 (line 105) |
| Setting | 5 | Describe the setting, locations, and relevant dates, including periods of recruitment, exposure, follow-up, and data collection | 5-6 (lines 105-126) |
| Participants | 6 | (*a*) Give the eligibility criteria, and the sources and methods of selection of participants | 6-7 (lines 127-121) |
| Variables | 7 | Clearly define all outcomes, exposures, predictors, potential confounders, and effect modifiers. Give diagnostic criteria, if applicable | 6-7 (lines 122-143) + supplemental Tables 1 & 2 |
| Data sources/ measurement | 8* | For each variable of interest, give sources of data and details of methods of assessment (measurement). Describe comparability of assessment methods if there is more than one group | 6-8 + Tables |
| Bias | 9 | Describe any efforts to address potential sources of bias | 8 line 174-176 |
| Study size | 10 | Explain how the study size was arrived at | 8 line 178-182 |
| Quantitative variables | 11 | Explain how quantitative variables were handled in the analyses. If applicable, describe which groupings were chosen and why | 8 line 183-191 |
| Statistical methods | 12 | (*a*) Describe all statistical methods, including those used to control for confounding | 7-8 line 183-191 |
|  |  | (*b*) Describe any methods used to examine subgroups and interactions | - |
|  |  | (*c*) Explain how missing data were addressed | 7-8 line 188-191 |
|  |  | (*d*) If applicable, describe analytical methods taking account of sampling strategy | - |
|  |  | (*e*) Describe any sensitivity analyses | - |
| Results | | | |
| Participants | 13* | (a) Report numbers of individuals at each stage of study—eg numbers potentially eligible, examined for eligibility, confirmed eligible, included in the study, completing follow-up, and analysed | 9 line 198-210 + Table 1 |
|  |  | (b) Give reasons for non-participation at each stage | - |
|  |  | (c) Consider use of a flow diagram | - |
| Descriptive data | 14* | (a) Give characteristics of study participants (eg demographic, clinical, social) and information on exposures and potential confounders | 9-11 & Table1 |
|  |  | (b) Indicate number of participants with missing data for each variable of interest | Table 1 |
| Outcome data | 15* | Report numbers of outcome events or summary measures | Table 2 |
| Main results | 16 | (*a*) Give unadjusted estimates and, if applicable, confounder-adjusted estimates and their precision (eg, 95% confidence interval). Make clear which confounders were adjusted for and why they were included | 11-15 & Tables 2 &3 |
|  |  | (*b*) Report category boundaries when continuous variables were categorized | Table 3 |
|  |  | (*c*) If relevant, consider translating estimates of relative risk into absolute risk for a meaningful time period | - |
| Other analyses | 17 | Report other analyses done—eg analyses of subgroups and interactions, and sensitivity analyses | - |
| Discussion | | | |
| Key results | 18 | Summarise key results with reference to study objectives | 20 lines 315-324 |
| Limitations | 19 | Discuss limitations of the study, taking into account sources of potential bias or imprecision. Discuss both direction and magnitude of any potential bias | 21-22 lines 334-356 |
| Interpretation | 20 | Give a cautious overall interpretation of results considering objectives, limitations, multiplicity of analyses, results from similar studies, and other relevant evidence | 22-23 |
| Generalisability | 21 | Discuss the generalisability (external validity) of the study results | 22-23 |
| Other information | | | |
| Funding | 22 | Give the source of funding and the role of the funders for the present study and, if applicable, for the original study on which the present article is based | 8 line 176-182 |

**Table S4:** STARD checklist NIDIAG study

|  | **Section & Topic** | **No** | **Item** | **Reported on page #** |
| --- | --- | --- | --- | --- |
|  |  |  |  |  |
|  | **TITLE OR ABSTRACT** |  |  |  |
|  |  | **1** | Identification as a study of diagnostic accuracy using at least one measure of accuracy  (such as sensitivity, specificity, predictive values, or AUC) | 1 |
|  | **ABSTRACT** |  |  |  |
|  |  | **2** | Structured summary of study design, methods, results, and conclusions  (for specific guidance, see STARD for Abstracts) | 2-3 |
|  | **INTRODUCTION** |  |  |  |
|  |  | **3** | Scientific and clinical background, including the intended use and clinical role of the index test | 4-5 |
|  |  | **4** | Study objectives and hypotheses | 5 (lines 100-102) |
|  | **METHODS** |  |  |  |
|  | *Study design* | **5** | Whether data collection was planned before the index test and reference standard  were performed (prospective study) or after (retrospective study) | 5 (line 105) |
|  | *Participants* | **6** | Eligibility criteria | 5-6 (lines 127-140) |
|  |  | **7** | On what basis potentially eligible participants were identified  (such as symptoms, results from previous tests, inclusion in registry) | 5-6 (lines 127-140) |
|  |  | **8** | Where and when potentially eligible participants were identified (setting, location and dates) | 5 -6 (lines 104-126) |
|  |  | **9** | Whether participants formed a consecutive, random or convenience series | 6 line 128-132 |
|  | *Test methods* | **10a** | Index test, in sufficient detail to allow replication | 7-8 line 165-173 |
|  |  | **10b** | Reference standard, in sufficient detail to allow replication | 7 line 141-164 |
|  |  | **11** | Rationale for choosing the reference standard (if alternatives exist) | Supplemental Table 1 |
|  |  | **12a** | Definition of and rationale for test positivity cut-offs or result categories  of the index test, distinguishing pre-specified from exploratory | 7-8 line 165-173 |
|  |  | **12b** | Definition of and rationale for test positivity cut-offs or result categories  of the reference standard, distinguishing pre-specified from exploratory | 7 line 141-164 & Supplemental Table 1 |
|  |  | **13a** | Whether clinical information and reference standard results were available  to the performers/readers of the index test | 8 line 173-176 |
|  |  | **13b** | Whether clinical information and index test results were available  to the assessors of the reference standard | 8 line 173-176 |
|  | *Analysis* | **14** | Methods for estimating or comparing measures of diagnostic accuracy | 8 line 183-191 |
|  |  | **15** | How indeterminate index test or reference standard results were handled8 line 183-191 | 8 line 183-191 |
|  |  | **16** | How missing data on the index test and reference standard were handled | 8 line 183-191 |
|  |  | **17** | Any analyses of variability in diagnostic accuracy, distinguishing pre-specified from exploratory | - |
|  |  | **18** | Intended sample size and how it was determined | 8 line 178-182 |
|  | **RESULTS** |  |  |  |
|  | *Participants* | **19** | Flow of participants, using a diagram | 9-11 |
|  |  | **20** | Baseline demographic and clinical characteristics of participants | 9-10 Table 1 + referring text |
|  |  | **21a** | Distribution of severity of disease in those with the target condition | Table 3 + referring text |
|  |  | **21b** | Distribution of alternative diagnoses in those without the target condition | Table 2 + referring text |
|  |  | **22** | Time interval and any clinical interventions between index test and reference standard | Page 6 and Supplemental Table 1 |
|  | *Test results* | **23** | Cross tabulation of the index test results (or their distribution)  by the results of the reference standard | Table 4A & 4B |
|  |  | **24** | Estimates of diagnostic accuracy and their precision (such as 95% confidence intervals) | Table 4A & 4B |
|  |  | **25** | Any adverse events from performing the index test or the reference standard | - |
|  | **DISCUSSION** |  |  |  |
|  |  | **26** | Study limitations, including sources of potential bias, statistical uncertainty, and generalisability | 21-22 lines 334-356 |
|  |  | **27** | Implications for practice, including the intended use and clinical role of the index test | 23-24 |
|  | **OTHER INFORMATION** |  |  |  |
|  |  | **28** | Registration number and name of registry | 24 line 419-425 |
|  |  | **29** | Where the full study protocol can be accessed | 4 line 86 |
|  |  | **30** | Sources of funding and other support; role of funders | 24 line 433-439 |
|  |  |  |  |  |
